# The role of mTORC1 in the regulation of skeletal muscle mass

**DOI:** 10.12703/r/11-32

**Published:** 2022-11-11

**Authors:** Sue C Bodine

**Affiliations:** 1 Department of Internal Medicine, Division of Endocrinology and Metabolism, University of Iowa Carver College of Medicine, 200 Hawkins Drive, Iowa City, IA 52242, USA

**Keywords:** atrophy, hypertrophy, protein synthesis, aging

## Abstract

Skeletal muscle mass is a very plastic characteristic of skeletal muscle and is regulated by signaling pathways that control the balance between anabolic and catabolic processes. The serine/threonine kinase mechanistic/mammalian target of rapamycin (mTOR) has been shown to be critically important in the regulation of skeletal muscle mass through its regulation of protein synthesis and degradation pathways. In this commentary, recent advances in the understanding of the role of mTORC1 in the regulation of muscle mass under conditions that induce hypertrophy and atrophy will be highlighted.

## Introduction

**Figure 1.**
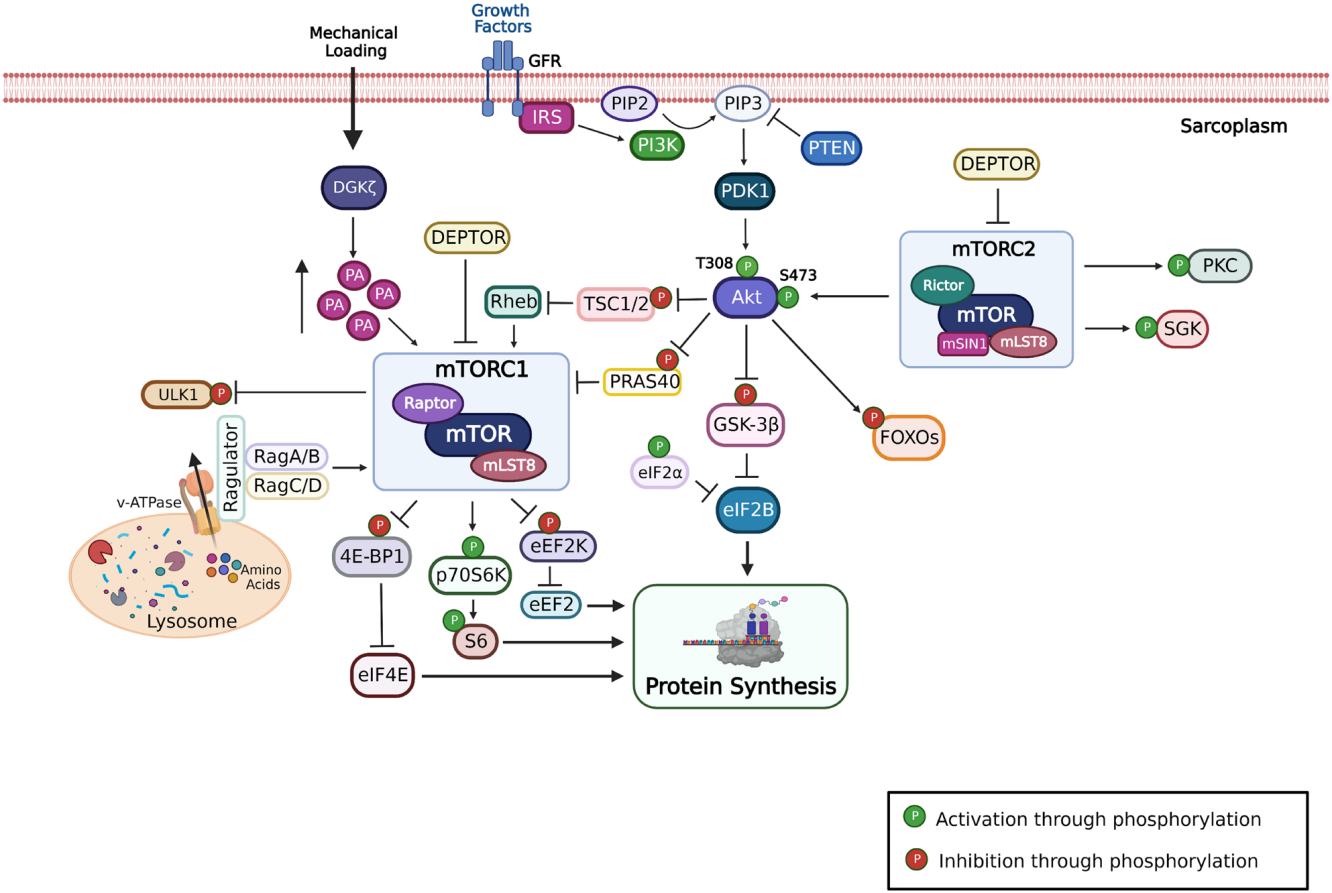
Schematic representation of the mTORC1 signaling pathway.

Skeletal muscle is a highly adaptive tissue that can modify its size throughout life in response to a variety of stimuli, including neural activity, external loading, growth factors, hormones, nutrients, inflammatory mediators, hypoxia, metabolic stress, and oxidative stress. Muscle mass is regulated primarily by signaling pathways that control the balance between protein synthesis and protein degradation. The serine/threonine kinase mechanistic/mammalian target of rapamycin (mTOR) exists in two functionally and structurally distinct protein complexes: the core components of mTORC1 are the regulatory-associated protein of TOR (raptor) and mammalian lethal with sec-13 protein 8 (mLST8), and the core components of mTORC2 are the rapamycin-insensitive companion of mTOR (RICTOR), mammalian stress-activated protein kinase-interacting protein 1 (mSIN1), and mLST8 (Figure 1)^1^. The mTORC1 complex has been shown to be responsive to multiple environmental signals, including nutrients (amino acids and glucose), mechanical load, growth factors (insulin-like growth factor 1, or IGF-1), hormones (insulin), and oxygen levels, suggesting that it plays a central role in the regulation of metabolism and growth^2^.

Manipulation of both upstream activators and downstream targets of mTORC1 in skeletal muscle has provided major advances in our understanding of the role of mTORC1 activity in the regulation of muscle mass and function (Figure 1). Investigation of the role of mTORC1 in the regulation of skeletal muscle size has been achieved through the use of pharmacological agents in humans, rats, and mice and genetic manipulation of selective genes in mouse skeletal muscles. The most common pharmacological approach has been systemic delivery of rapamycin since mTORC1 is rapamycin-sensitive and mTORC2 is rapamycin-insensitive; however, chronic delivery of high doses of rapamycin can also block mTORC2. The mTOR kinase inhibitor, AZD8055, has also been used to block both mTORC1 and mTORC2 activation. Genetically, mTORC1 can be selectively inhibited through the deletion of raptor while mTORC1 can be chronically activated through the deletion of TSC1. Pharmacological and genetic approaches have produced both complementary and variable results. The variable results may relate to the fact that mTORC1 activity is not completely blocked by systemically delivered rapamycin while genetic deletion of raptor produces a more complete block of mTORC1 activation.

In skeletal muscle, mTORC1 signaling has been shown to be an important regulator of muscle size through its regulation of mRNA translation and likely other processes such as autophagy and metabolism^1^. The major downstream targets of mTORC1 are p70^s6K^ (S6K1), 4E-BP1, eIF2k, and ULK-1 (Figure 1). The important role of mTORC1 activation in the regulation of skeletal muscle size has emerged over the past two decades. Early genetic manipulation studies in *Drosophila* of PI(3)K, PKB/Akt, and p70^s6k^ revealed that deletion of components of this pathway resulted in smaller cells but not fewer cells, suggesting that the PI(3)K/Akt/mTOR pathway played a critical role in the regulation of cell size^3^. Further genetic manipulations of the PI(3)K/Akt/mTOR pathway in mice revealed the importance of this pathway in the early growth and development of skeletal muscle^4^; however, little was known about the role of this pathway in the maintenance and growth of skeletal muscle in adult mammals. The first suggestion that activation of mTORC1 was important for muscle hypertrophy came from Baar and Esser^5^, who reported that an acute bout of resistance exercise-like lengthening contractions induced an increase in the phosphorylation of p70^s6k^ (S6K1) in rat skeletal muscle and that the magnitude of the increase in p70^s6k^ phosphorylation was correlated with the degree of hypertrophy following a 6-week training protocol. These findings were extended by Bodine *et al.*^6^, who demonstrated that the Akt/mTOR/p70^s6k^/4E-BP1 pathway was activated in the plantaris muscle following synergist ablation, or functional overload, leading to muscle hypertrophy. Moreover, it was demonstrated that systemic administration of rapamycin at a dose of 1.5 mg/kg per day could suppress phosphorylation of mTOR, p70^s6k^, and 4E-BP1 and, importantly, prevent hypertrophy of slow and fast fibers in the rat plantaris following functional overload^6,7^. Bodine *et al*.^6^ also showed that the Akt/mTOR pathway was suppressed in response to hindlimb unloading that resulted in muscle fiber atrophy and reactivated upon hindlimb reloading, which induced muscle fiber growth. Rapamycin treatment given upon reloading of the hindlimbs significantly reduced the regrowth of the soleus, plantaris, and medial gastrocnemius muscles; however, the growth inhibition was only about 50%, suggesting the involvement of additional anabolic pathways^6^. These initial observations have been confirmed and extended by multiple independent groups, showing that activation of mTORC1 is necessary and sufficient to induce muscle hypertrophy^8,9^.

Although there have been considerable investigations of the role of mTORC1 activation in the induction of muscle growth since the early 2000s, the exploration of the role of mTORC1 activity in the induction of muscle atrophy has been explored only recently. Several excellent reviews have summarized the current state of knowledge regarding the regulation of mTOR signaling and its control of metabolism in multiple tissues^1,2^. In this commentary, the focus will be on recent advances in the understanding of the role of mTORC1 in the regulation of skeletal muscle size in adult animals.

## mTORC1 signaling and the maintenance of adult skeletal muscle mass

The prevailing evidence suggests that activation of mTORC1 is necessary for proper development and postnatal growth of skeletal muscle; however, the need for mTORC1 activation in the maintenance of muscle mass in mature, adult animals has been unclear. Previous reports have shown that selective deletion of mTOR or raptor in skeletal muscle during embryonic development leads to a reduction in postnatal growth and the development of late-onset myopathy and premature death around 6 to 8 months of age^10,11^. In contrast, inducible-skeletal muscle-specific deletion of raptor in young adult mice for 21 days did not induce skeletal muscle atrophy^12^. The results from genetically manipulated mice are consistent with the observation that rapamycin treatment in young adult rats (10–12 weeks old) did not affect muscle mass but that rapamycin treatment for 14 days initiated in 2-week-old rat pups resulted in a 40% decrease in the hindlimb muscle mass^13^. Recently, Ham *et al*.^14^ induced the deletion of raptor in the muscles of 3-month-old mice and examined the effect at different time points (10 days, 21 days, and 5 months) after deletion. Raptor deletion suppressed the phosphorylation of downstream mTORC1 targets but had no effect on hindlimb muscle weight or maximum isometric force production 5 months after deletion^14^. After 5 months of raptor deletion, however, an increase in fiber size variability was observed within individual muscles, with the appearance of very small and very large fibers but no change in mean fiber cross-sectional area. Furthermore, a 24% reduction in ribosomal proteins and a reduction in the percentage of heavy ribosomes were reported, suggesting a decrease in translational capacity and global translation^14^. These data are consistent with the 40% reduction in basal protein synthesis measured by puromycin reported by You *et al*.^12^ following 21 days of induced raptor deletion in adult mice. Additionally, acute *in vivo* treatment of adult rats with either rapamycin (inhibition of mTORC1 activity only) or the mTOR kinase inhibitor AZD8055 (inhibition of TORC1 and mTORC2 activity) resulted in a 40 to 50% decrease in basal protein synthesis in hindlimb muscles^15^. These results suggest that, in mature adult animals, a significant portion of basal protein synthesis is mTORC1-independent. Furthermore, the results suggest that maintenance of adult muscle is not solely dependent on mTORC1 activity; however, the long-term suppression of mTORC1 may not be without some negative consequences to muscle, one being an inability to positively respond to anabolic signals with an increase in muscle size and strength.

## mTORC1 signaling and increases in protein synthesis following mechanical stimulation

Skeletal muscle hypertrophy occurs in adult animals as the result of repeated increases in mechanical loading, and the precise role of mTORC1 activation in stimulating an increase in protein synthesis and muscle fiber size continues to be investigated. In humans, resistance exercise training is known to produce increases in muscle size and strength, as well as protein synthesis^16^. In rodents, resistance exercise has been simulated using several models, the most common being functional overload (or synergist ablation) and electrical stimulation (ES). Functional overload is most often performed on the plantaris muscle through the removal of the soleus and the distal half of the medial and lateral gastrocnemius muscle. Functional overload induces a chronic increase in external loading as well as an increase in the neural activation of the plantaris muscle, resulting in a rapid increase in muscle mass and fiber cross-sectional area. Bodine *et al*.^6^ revealed that muscle growth in response to the functional overload of the rat plantaris was rapamycin-sensitive, thus suggesting that it was mTORC1-dependent. Follow-up studies have been performed in genetically modified C57BL6 mice, confirming that mTORC1 is activated in the muscle fiber in response to mechanical loading and required to induce muscle growth following functional overload^17^. The functional overload model in C57BL6 mice, but not rats, results in an increase in the number of fibers within the plantaris and an increase in the expression of embryonic myosin heavy chain, suggestive of degeneration/regeneration or *de novo* myogenesis or both. In more recent studies, a modified functional overload model, referred to as myotenectomy, was developed in which the soleus muscle was left intact and only the distal tendon of the gastrocnemius muscle was removed in order to investigate the role of mTORC1 in muscle fiber growth in response to mechanical overload. With a muscle-specific, tamoxifen-inducible raptor knockout (KO) mouse, growth of the plantaris muscle was found to be completely blocked in response to mechanical overload induced by myotenectomy^12^. Interestingly, the mechanical overload-induced increase in protein synthesis, as measured by puromycin, was not blocked-in raptor KO mice or rapamycin (0.6 mg/kg)-treated wild-type mice^12^. For comparison, 7 days of rapamycin (1.5 mg/kg) treatment in young female FVB/N mice was found to inhibit p70^s6K ^phosphorylation, muscle growth, and the increase in protein synthesis as measured by puromycin following myotenectomy^18^. The contradictory findings related to the suppression of protein synthesis following myotenectomy could be related to the rapamycin doses used in the two studies (0.6 vs. 1.5 mg/kg) and the potential inhibition of 4E-BP1. A rapamycin dose of 1.5 mg/kg was found to suppress both p70^s6K^ phosphorylation and 4E-BP1 bound to eIF4E, as well as muscle growth following 14 days of functional overload in the rat plantaris^6^.

While functional overload has been an extremely useful model for identifying the cellular and molecular mechanisms underlying skeletal muscle growth, it does induce a very rapid hypertrophy response, which is not typical of resistance exercise in humans. A rodent model that may better simulate human resistance exercise is repeated ES. In humans, it has been shown that an acute bout of resistance exercise activates mTORC1 and its downstream targets and that systemic delivery of rapamycin blocks the increase in protein synthesis and partially blocks the activation of downstream mTORC1 targets such as p70^S6K^^19^. In rodents, repeated maximal ES of the sciatic nerve has been used to simulate resistance exercise. In 2019, You *et al*.^12^ demonstrated that mTORC1 signaling increases in response to a single bout of ES and can be blocked by rapamycin or deletion of raptor. Furthermore, raptor was found to be necessary for the targeting of mTOR to the late endosome/lysosome in response to ES^12^. In response to a single bout of resistance exercise, protein synthesis can be elevated for up to 48 hours after exercise, and several recent studies have examined the extent to which this elevated protein synthesis is rapamycin-sensitive/mTORC1-dependent. Two independent studies used slightly different resistance exercise models in rats and found that rapamycin blocked the early increase in protein synthesis but failed to completely block the late increase in protein synthesis induced by an acute bout of exercise^20,21^. In a follow-up study, Ogasawara and Suginohara^15^ showed that the ATP-competitive mTOR kinase inhibitor, AZD8055, inhibited mTORC1 activation and the phosphorylation of Akt at Ser 473 and completely inhibited the resistance exercise-induced increase in protein synthesis. In Ogasawara *et al*.^21^, chronic rapamycin treatment (1.5 mg/kg) was found to significantly reduce, but not fully suppress, the increase in plantaris fiber cross-sectional area in response to 4 weeks of chronic resistance exercise.

In summary, these recent studies reveal that both mTORC1-dependent and mTORC1-independent pathways are activated in response to mechanical loading and contribute to increases in protein synthesis. The data are consistent with the conclusion that activation of mTORC1 is a critical, if not necessary, step for the induction of muscle growth in response to mechanical loading. Recent evidence reveals that mTORC1 is not chronically active in response to repeated bouts of resistance exercise, and, in fact, activation of mTORC1 following repeated bouts of resistance exercise may be reduced relative to the strong activation that occurs following the initial bout of exercise^22^. These recent studies highlight the need for further investigations into the mTORC1-independent pathways that are activated in response to resistance exercise and appear to contribute to an increase in protein synthesis. Furthermore, details of the specific proteins that are translated by mTORC1-dependent and mTORC1-independent pathways are needed.

## mTORC1 signaling and muscle atrophy

A common misconception in the literature is that, under conditions that induce muscle atrophy, protein synthesis is suppressed and, as an extension, that mTORC1 signaling is suppressed. It is true that protein synthesis is inhibited under many atrophy-inducing conditions, such as sepsis, fasting, diabetes, and hindlimb unloading^23^. However, under conditions of cast immobilization and denervation, mTORC1 activity has been reported to increase, not decrease, at least during the early phases of the atrophy process^24,25^. These findings raise several questions: What are the upstream activators of mTORC1? What is the purpose of the increase in mTORC1 activity? Is an increase in mTORC1 protective or harmful? It is not unreasonable to think that the synthesis of selective proteins could increase in response to an atrophy-inducing event, especially given the large number of genes that are transcriptionally upregulated following atrophy-inducing conditions, such as immobilization and denervation. In this case, the mRNA species being translated could be supporting protein degradation pathways and thus be contributing to the atrophy process.

Several studies have found that joint immobilization results in an increase in mTORC1 activity, as measured by an increase in p70^s6k^ phosphorylation and cap-dependent translation^25,26^. This increase in mTORC1 activity, however, is associated with a decrease in protein synthesis. Recently, Lin *et al*.^27^ reported a significant decrease in protein synthesis in the plantar flexor muscles of male mice after 6 hours of rigid immobilization of the ankle and knee joints, which continued to decline for 72 hours. The lack of suppression of the mTORC1 pathway following immobilization differs from what is observed following hindlimb unloading, where there is a decrease in protein synthesis and mTORC1 activity, especially in the soleus^28^. The difference between joint immobilization and hindlimb unloading is unclear but could relate to the extent to which neural activation of the muscles is affected in the two models; immobilization produces a greater and more sustained decrease in neural activity than hindlimb unloading^29,30^.

To gain insight into the role of increased mTORC1 activity, rapamycin was delivered to mice during the immobilization period and found to exacerbate the immobilization-induced decrease in protein synthesis and the loss of muscle mass and fiber cross-sectional area^25^. Interestingly, further activation of mTORC1 and protein synthesis in the immobilized muscle with overexpression of Rheb resulted in attenuated atrophy^25^. It was concluded, on the basis of these data, that the increase in mTORC1 activity during immobilization was protective in that it prevented the further decline in protein synthesis. In a more recent study, Segalés *et al*.^31^ examined the effect of sestrin 1 and 2 overexpression on mTORC1 activity and muscle atrophy. The overexpression of sestrin 1 or sestrin 2 was able to prevent the loss of muscle mass and function following limb immobilization and denervation through proposed mechanisms of action involving inhibition of mTORC1 and activation of mTORC2, resulting in the inhibition of ubiquitin-proteasome-mediated proteolysis and the activation of autophagy^31^. Interestingly, they reported that rapamycin treatment resulted in the sparing of muscle mass following immobilization^31^. The apparent contradictory effects of mTORC1 inhibition may be related to the dose of rapamycin used (4 vs. 1.5 mg/kg), which could have differentially affected protein degradation since You *et al*.^25^ reported no effect of rapamycin treatment on protein degradation whereas Segalés *et al*.^31^ reported inhibition of FOXO-mediated upregulation of ubiquitin-proteasome proteolysis and an increase in autophagy.

As mentioned, denervation-induced muscle atrophy also results in an increase in mTORC1 activity as well as increases in Akt phosphorylation and protein synthesis^24,32^. Tang *et al*.^24^ suggested that activation of mTORC1 was deleterious in that it inhibited Akt phosphorylation, resulting in the activation of the FOXO transcription factors. However, increased Akt phosphorylation has been shown to be an early response to denervation as opposed to a late response^33^. Furthermore, an increase in the transcription, translation, and activity of FOXO transcription factors occurs early following denervation, even with an increase in Akt phosphorylation, and deletion of the FOXO transcription factors (FOXO1/3/4) protects against denervation-induced muscle atrophy^34^.

Previous studies in mice^33^ and rats^35^ have reported that rapamycin treatment does not protect from denervation-induced atrophy, nor does it exacerbate muscle loss. However, recent studies performed in muscle-specific raptor KO mice suggest that the activation of mTORC1 activity in response to denervation is protective since raptor KO mice have greater denervation-induced atrophy than wild-type mice^36^. Interestingly, measurement of protein synthesis in specific fiber types within the fast-twitch extensor digitorum longus (EDL) muscle revealed that protein synthesis selectively increased in the non-type IIb fibers (that is, the more oxidative fibers) following denervation and that only the non-type IIb fibers in the EDL and tibialis anterior (TA) muscles had greater atrophy in the raptor KO mice, suggesting that the mTORC1 protection is fiber type-specific^36^. The findings from the raptor KO mice suggest that activation of mTORC1 in response to denervation serves to protect the muscle from severe atrophy; however, mice with chronic activation of mTORC1 (TSC1mKO) also show more severe atrophy following denervation, especially of types IIA/X and I fibers, and denervation for an extended period (4 weeks) produces a severe myopathy similar to what is observed in 9- to 10-month-old TSC1mKO mice^37^. The more severe atrophy response was attributed to a blunted increase in Akt phosphorylation, a dysregulated autophagy response, and blunting of HDAC4 activation.

These recent findings show the complexity of mTORC1 regulation and highlight the need to better understand the role of mTORC1 activity under atrophy-inducing conditions. Both increases and decreases in mTORC1 activity have been associated with muscle atrophy induced by disuse. Moreover, increases in mTORC1 activity under disuse atrophy conditions have been associated with both increases and decreases in protein synthesis. In this regard, the extent to which changes in mTORC1 activity are responsible for the changes in global protein synthesis is unclear. Going forward, we need a better understanding of the translation and degradation of specific proteins under different atrophy conditions. One must also be cognizant of the impact of muscle type and atrophy duration on the findings, as well as the rodent species (mouse versus rat) under study and its age and sex.

## mTORC1, the neuromuscular junction, and aging

A preponderance of evidence supports the critical role of mTORC1 activity in the formation and maintenance of the neuromuscular junction (NMJ) in mammals^38^. Interestingly, both the prolonged inhibition of mTORC1 through inducible muscle-specific deletion of raptor and the chronic activation of mTORC1 through the muscle-specific deletion of TSC1 have been shown to lead to NMJ destabilization. In the case of mTORC1 inhibition, Baraldo *et al*.^39^ found that 7 months following raptor deletion in adult mice, there was an increase in the percentage of neural cell adhesion molecule (NCAM)-positive fibers and fibrillation potentials and an increase in endplate fragmentation that suggested the presence of denervation. Interestingly, only minimal changes in body weight and muscle mass were found; however, an increase in fiber size variability with the appearance of small, angulated fibers was observed in specific muscles^39^. The suggested causes of the NMJ fragmentation and denervation in mice with prolonged mTORC1 inhibition were a block in autophagic flux and mitophagy leading to mitochondrial dysfunction^39^. The effects of long-term suppression of mTORC1 through the deletion of raptor in adult muscle could reflect the importance of mTORC1 in the regulation of metabolism and other cellular processes.

In comparison, chronic activation of mTORC1 in TSC1mKO mice also results in endplate fragmentation as well as a reduction in acetylcholine receptor density and impaired neural transmission^37^. In contrast to the raptor KO mice, TSC1mKO mice develop early-onset muscle atrophy followed by a severe late-stage myopathy characterized by vacuolated fibers. Prolonged activation of mTORC1 in the TSC1mKO mice also inhibits Akt phosphorylation, resulting in the activation of FOXO transcription factors and the upregulation of the E3 ligases MuRF1 and MAFbx/atrogin1 in some muscles, which may contribute to the early (3 months) atrophy, while the late-stage myopathy was linked to the suppression of autophagy and further NMJ fragmentation and denervation^37^.

There is growing evidence that mTORC1 activity is chronically elevated in skeletal muscle with aging and may contribute to sarcopenia^40,41^. The upstream factors contributing to the activation of mTORC1 with age are unknown, but it has been speculated that denervation contributes to the elevation in basal mTORC1 activity. Multiple factors have been suggested to contribute to sarcopenia, including altered proteostasis, inflammation, and NMJ instability, which could be influenced by changes in mTORC1 activity. In this regard, several studies have examined the impact of rapamycin or rapamycin analogs (rapalogs) on the progression of sarcopenia^40,41^. Low-dose rapalog treatment, started at 22 months of age for 6 weeks in Sprague Dawley rats, was able to prevent the loss of mass in some (TA and plantaris) but not all (gastrocnemius) muscles^40^. Low-dose rapalog treatment also suppressed the expression of selected atrophy-associated genes and inactivity/denervation-associated genes in selected muscles. A recent study in mice examined the impact of rapamycin (4 mg/kg) treatment started at 15 or 20 months of age and followed until 30 months of age^42^. As seen in the rat, rapamycin treatment was differentially effective in preventing loss of muscle mass and strength. Differential gene expression analysis in multiple muscle types revealed that rapamycin treatment had significant effects on multiple cellular processes, including immune responses, NMJ, and the extracellular matrix^42^. The suppression of mTORC1 remains a potential target for aging; however, future studies will need to identify the optimal dose and dosing strategy if it is to remain a viable therapeutic. Future studies should also examine the effect of chronic mTORC1 inhibition on the adaptive responses to endurance and resistance exercise.

## Concluding remarks

The accumulating evidence clearly shows that mTORC1 is an important regulator of skeletal muscle mass and function. However, the role of mTORC1 activity in skeletal muscle is complex and dependent on the life stage (that is, postnatal versus adult versus old). The published data suggest that mTORC1 activity is tightly regulated throughout the lifespan and is responsive to a variety of external stimuli. However, the mechanisms by which these external signals activate or inhibit mTORC1 are unclear. In general, the data suggest that acute, intermittent elevations in mTORC1 activity are beneficial; however, chronic elevations in mTORC1 activity in adult muscles appear to be detrimental.

Some key points are the following:

1. During postnatal development, elevated levels of mTORC1 activity are necessary for the growth of muscle fibers. However, in adult mammals, resting mTORC1 activity is reduced and does not appear to be necessary for the maintenance of muscle mass since the suppression of resting mTORC1 activity in adult muscle by rapamycin or raptor deletion does not induce muscle loss but does cause a slight reduction in basal protein synthesis.
2. Although the complete inhibition of mTORC1 activation by the deletion of raptor in adult muscles does not induce muscle atrophy in the short term, extended inhibition of mTORC1 does lead to muscle dysfunction which could be related to the fact that mTORC1 activity influences not only growth processes but other cellular processes involved in metabolism, glucose homeostasis, and proteostasis.
3. Although activation of mTORC1 may not be required for the maintenance of muscle mass in adult animals, it does appear to be a critical pathway for the induction of adaptive muscle growth, especially in response to mechanical overload. In response to a bout of resistance exercise, there is an increase in both mTORC1 activity and protein synthesis. Increases in mTORC1 activity appear to be responsible for some but not all of the increases in protein synthesis. It appears that both mTORC1-dependent and mTORC1-independent pathways are involved in the control of protein synthesis and the regulation of muscle mass.
4. The role of mTORC1 activity in regulating the loss of muscle mass is unclear. It appears that the suppression of mTORC1 alone does not induce muscle loss, at least in the short term. It remains unclear as to whether the elevated mTORC1 activity observed during disuse or denervation is beneficial or detrimental.
5. Several investigations have shown that activation of mTORC1 through Akt activation or other mechanisms can attenuate the loss of muscle mass under a variety of conditions^6,35,43,44^.
6. Finally, whereas intermittent activation of mTORC1 is critical for inducing muscle hypertrophy, chronic activation of mTORC1 is harmful, leading to muscle atrophy and dysfunction. Accumulating evidence suggests that resting mTORC1 activity is chronically elevated in skeletal muscle as a function of age and may lead to dysfunction, especially at the NMJ.

Although significant advancements in our understanding of the function of mTORC1 activity in muscles have been made, many questions remain to be answered. For example:

1. What are the specific upstream factors that lead to activation or suppression of mTORC1 activity under atrophy conditions?
2. What is the role of increased mTORC1 following denervation or immobilization? Is it beneficial or harmful? Is increased mTORC1 regulating the translation of specific proteins? If so, what is the mechanism for the translation of selective mRNA species?
3. Can activation of mTORC1 activity alone attenuate muscle atrophy? If not, by what mechanism does mTORC1 need to be activated in order to attenuate muscle atrophy? Is activation of Akt necessary to attenuate muscle atrophy?
4. How is mechanical load sensed and how is it transduced to activate mTORC1?
5. What mTORC1-independent pathways are responsible for the maintenance of muscle mass in adult animals?
6. What is responsible for the chronic activation of mTORC1 with aging? What is the relationship between mTORC1 activation with age and the development of sarcopenia?
7. Is long-term pharmacological suppression of mTORC1 activation harmful to skeletal muscle? Does chronic suppression of mTORC1 with rapamycin suppress muscle adaptation to endurance or resistance exercise?
